# The role of the apoptosis-related protein BCL-B in the regulation of mitophagy in hepatic stellate cells during the regression of liver fibrosis

**DOI:** 10.1038/s12276-018-0199-6

**Published:** 2019-01-11

**Authors:** Qian Ding, Xiao-Li Xie, Miao-Miao Wang, Jie Yin, Jin-Mei Tian, Xiao-Yu Jiang, Di Zhang, Jing Han, Yun Bai, Zi-Jin Cui, Hui-Qing Jiang

**Affiliations:** Department of Gastroenterology, The Second Hospital of Hebei Medical University, Hebei Key Laboratory of Gastroenterology, Hebei Institute of Gastroenterology, Shijiazhuang, Hebei, China

**Keywords:** Liver fibrosis, Apoptosis

## Abstract

The clearance of activated hepatic stellate cells (HSCs) by apoptosis is critical for the reversibility of hepatic fibrosis. Mitochondrial homeostasis is regulated by mitophagy, which is an efficient way of clearing injured mitochondria that plays an important role in apoptosis. However, the role of mitophagy in apoptosis in HSCs and hepatic fibrosis is still unclear. Here, we show that mitophagy is enhanced in parallel with increased apoptosis in hepatic stellate cells during the reversal of hepatic fibrosis. The inhibition of mitophagy suppressed apoptosis in HSCs and aggravated hepatic fibrosis in mice. In contrast, the activation of mitophagy induced apoptosis in HSCs. Furthermore, we confirmed that BCL-B, which is a member of the BCL-2 family, is a regulator mediating mitophagy-related apoptosis. The knockdown of BCL-B resulted in increased apoptosis and mitophagy in HSCs, while the overexpression of BCL-B caused the opposite effects. BCL-B inhibited the phosphorylation of Parkin (a key regulator of mitophagy) and directly bound phospho-Parkin. Altogether, enhanced mitophagy promotes apoptosis in HSCs during the reversal of hepatic fibrosis. BCL-B suppresses mitophagy in HSCs by binding and suppressing phospho-Parkin, thereby inhibiting apoptosis. BCL-B-dependent mitophagy is a new pathway for the regulation of apoptosis in HSCs during the regression of hepatic fibrosis.

## Liver fibrosis: cleaning up injured mitochondria helps reverse tissue scarring

Clearing away defective mitochondria helps destroy cells in the liver that contribute to tissue scarring; the signaling pathway involved offers a new therapeutic target. Hui-Qing Jiang and colleagues from the Hebei Institute of Gastroenterology in Shijiazhuang, China, induced liver fibrosis in mice and showed that as the animals recovered and the damage to their liver tissue was reversed, injured mitochondria were cleared from fibrosis-causing cells in tandem with the cells’ controlled destruction. Experimentally inhibiting the process of mitochondrial clearance also inhibited cell death and aggravated fibrotic scarring in the mice. The researchers identified a signaling pathway that regulates mitochondrial cleanup and, in turn, also controlled cell death. Targeting this pathway offer a potential new therapeutic strategy for reversing liver fibrosis in patients.

## Introduction

Hepatic fibrosis is an outcome of many chronic liver diseases that is characterized by the excessive deposition of extracellular matrix proteins, especially collagen I, in the liver^1^. The activation of hepatic stellate cells (HSCs) is a key event in hepatic fibrosis since activated HSCs are the major source of collagen I^2^. HSCs undergo apoptosis during fibrosis reversal^3^. Therefore, the clearance of activated HSCs by apoptosis is critical for the reversibility of fibrosis^4^.

There are two major apoptosis signaling pathways, i.e., the extrinsic pathway and the intrinsic pathway. The intrinsic apoptotic pathway, which is also called the mitochondrial apoptotic pathway, is triggered by mitochondrial outer membrane permeabilization (MOMP) in response to intracellular stressors. MOMP, which is regulated by members of the BCL-2 family, stimulates the formation of the apoptosome, which, in turn, results in cell death^5^.

In addition to activating the intrinsic apoptosis pathway, MOMP plays an important role in mitophagy, which is a broad term used to refer to the selective degradation of mitochondria in lysosomes via a specific autophagic pathway^6^. Mitophagy is an efficient way of clearing injured mitochondria to maintain mitochondrial homeostasis^7^. Mitophagy is regulated by various factors, and the PINK1/Parkin pathway is considered the most important regulatory pathway. PINK1 recruits and activates Parkin’s E3 ubiquitin ligase activity, which, in turn, initiates mitophagy^8^. Research investigating mitophagy in liver disease has mainly focused on hepatocytes. Parkin-mediated mitophagy has been reported to occur in hepatocytes after treatment with APAP^9^, and Parkin protects against alcohol-induced liver injury and steatosis via mitophagy and the maintenance of mitochondrial function^10^. However, limited research investigating mitophagy in HSCs and its effect on hepatic fibrosis has been performed.

It is well-known that BCL-2 family proteins are major regulators of apoptosis^11^. Growing evidence suggest that the BCL-2 family members perform other functions in addition to their canonical role in apoptosis, including mitophagy^12^. BCL-B (also called BCL2L10, Boo and Diva) is the most recently discovered member of the human Bcl-2 family and is localized to the mitochondria^13^. Although BCL-B was initially identified as an anti-apoptotic protein^14^, it exerts a pro-apoptotic effect on some cancer cells^15,16^. It is possible that BCL-B performs different functions depending on the cellular context. As a member of the Bcl-2 family, BCL-B has been reported to influence mitophagy in HeLa cells^17^. However, the effects of BCL-B on HSC apoptosis and mitophagy are still unclear.

The aim of the present study is to investigate the role of mitophagy in HSCs during the reversal of liver fibrosis and identify the key factor linking mitophagy and apoptosis in HSCs during the regression of hepatic fibrosis.

## Materials and methods

### Liver fibrosis recovery model

Male C57BL/6 mice (6–8 weeks) were purchased from Beijing Vital River Laboratory Animal Technology Co., Ltd. (Beijing, China). The experimental models of reversible fibrosis were established by intraperitoneal injections of CCl_4_ diluted 1:9 in olive oil at a dosage of 5 μL/g bodyweight twice a week for 6 weeks. Olive oil injections served as a control. Livers and serum from the mice were harvested at peak fibrosis (immediately after the final injection of CCl_4_) and after 1, 2 and 4 weeks of spontaneous recovery (*n* = 6 per time point). This study was performed via a protocol approved by the Institutional Animal Care and Use Committee of Hebei Medical University in accordance with the Guide for the Care and Use of Laboratory Animals.

### Serum aminotransferase activity

The serum activities of alanine aminotransferase (ALT) and aspartate aminotransferase (AST) were detected using ALT and AST assay kits according to the manufacturer’s protocol (Jiancheng Bioengineering Institute, Nanjing, China).

### Histological and immunohistochemical analyses

The livers were fixed with 4% paraformaldehyde for 48 h, embedded in paraffin, and sliced into 4 μm sections. Then, the sections were stained with H&E for the morphological evaluation and Masson stain or Sirius-red stain for the assessment of collagen. The collagen I (1:50, Affinity) and α-SMA (1:200, Abcam) immunohistochemical staining was performed following heat-induced epitope retrieval as previously described^18^.

### Isolation and culture of primary hepatic stellate cells

HSCs were isolated from C57BL/6 mice as previously described^19,20^. Briefly, after perfusing the livers with collagenase and pronase (Gibco BRL, Gathersburg, MD, USA), the primary HSCs were isolated by Percoll density gradient centrifugation. The HSCs were subsequently cultured in Dulbecco’s Modified Eagle Medium/Nutrient Mixture F-12 (DMEM/F-12; Gibco, Carlsbad, CA, USA) supplemented with 20% fetal bovine serum (FBS; Life Technologies, Carlsbad, CA, USA). After 24 h of incubation, the medium was replaced with DMEM/F-12 supplemented with 10% FBS. After 3 d in culture, the cells were harvested for the subsequent experiments.

### HSC-LX2 cell line

The human HSC cell line LX2 was cultured in Dulbecco’s-modified Eagle’s medium (DMEM; Gibco BRL, Rockville, MD, USA) supplemented with 10% FBS. The HSCs were serum starved for 24 h and divided into the following treatment groups: apoptosis inducer gliotoxin (GTX, Abcam, Cambridge, UK) (1 μM) for 4 h, mitophagy activator valinomycin (Val, Cayman, USA) (2 μM) for 12 h, mitophagy inhibitor cyclosporine A (CsA, Cayman, USA) (10 μM) for 24 h, and GTX (1 μM) + CsA (10 μM). All experiments were repeated at least three times.

### Adenovirus and siRNA transfection

All GV314 (CMV-MCS-3FLAG-SV40-EGFP) adenovirus vectors were purchased from GeneChem (Shanghai, China). The LX-2 cells were transfected with an adenovirus carrying the BCL-B gene (adBCL-B) or a control adenovirus (adCon) for 8–12 h. The cells were harvested 48 h after transfection. The efficiency was tested by western blotting.

All small-interfering RNAs (siRNAs) were purchased from GenePharma (Suzhou, China). The LX-2 cells were transfected with either siRNA targeting BCL-B (si-BCL-B) or scrambled control siRNA (si-Con) using Lipofectamine2000 (Invitrogen, Carlsbad, CA, USA) for 6 h according to the protocol. Then, the medium was changed, and the cells were incubated for an additional 48 h. The efficiency was tested by western blotting.

### Western blot analysis

The liver tissues and LX2 cells were washed, homogenized on ice with RIPA buffer (Sigma-Aldrich, Saint Louis, MO, USA), and centrifuged at 8000 rpm for 10 min. The protein concentration in the supernatant was determined using a Bradford assay. Lysates containing equal amounts of protein were separated by SDS-PAGE. The western blot analysis was performed as previously described^21^. The following primary antibodies were used in this study: rabbit anti-BCL2L10 (1:500, Origene), rabbit anti-glyceraldehyde phosphate dehydrogenase (GAPDH; 1:8000, Cell Signaling Technology), rabbit anti-cleaved caspase3 (1:400, Affinity), rabbit anti-cleaved caspase9 (1:400, Affinity), rabbit anti-collagen I (1:700, Affinity), rabbit anti-α-smooth muscle actin (α-SMA; 1:1000, Abcam), rabbit anti-TOM20 (1:2000, Affinity), and mouse anti-BCL2L10 (1:400, Abcam). After incubating with fluorescence-conjugated secondary antibodies (1:20000, Rockland Biochemicals), the immunoreactive bands were visualized using an Odyssey Infrared Imaging System (LI-COR Biosciences). For the protein quantification, the bands were scanned and quantified using Image-Pro plus 6.0 software (Datacell, UK), and GAPDH served as an internal control.

### Apoptosis assay

Following the treatment, the cells (2 × 10^5^/sample) were harvested and washed twice with precooled PBS. An Alexa Fluor488 annexinV/Dead Cell Apoptosis Kit and Alexa Fluor488 annexinV and PI for flow cytometry kit (Invitrogen, Carlsbad, CA, USA) was utilized for the detection of apoptotic cells. Briefly, 5 μL aliquots of Annexin V and 1 μL aliquots of propidium iodine (PI) buffer were added to 400 μL of binding buffer. Then, the cells were exposed to the mixed solution for 15 min in the dark at room temperature. The samples were analyzed with fluorescence-activated cell sorting (FACS; Beckman-Coulter, Fullerton, CA, USA). The percentage of Annexin V positive cells was recorded as a measurement of cell apoptosis.

In addition, the terminal deoxynucleotidyl transferase-mediated deoxyuridine triphosphate nick-end labeling (TUNEL) technique was employed for the detection of apoptosis in the cells. The staining steps were performed according to the instructions provided by the TUNEL and Fluor™ 594 apoptosis detection kit (GeneCopoeia, USA). The apoptosis index was calculated as the percentage of TUNEL-positive cells that showed clear purple nuclear staining.

### Co-immunoprecipitation

The lysate samples were precleared with Protein A/G PLUS-Agarose (Santa Cruz, CA, USA) beads to reduce nonspecific binding. The supernatants were immunoprecipitated with the indicated antibodies at 4 °C for 2 h, followed by incubation with Protein A/G PLUS-Agarose beads overnight. Then, the agarose beads were collected by centrifugation, washed with lysis buffer, and resuspended in sample buffer. The bound proteins were resolved by SDS-PAGE, followed by a western blot analysis as described above.

### Double-immunofluorescence assay

LX-2 cells grown on coverslips were fixed in 4% paraformaldehyde for 20 min, permeabilized with PBS supplemented with 0.1% Triton X-100 for 10 min and blocked using 10% normal goat serum in PBS for 30 min. Then, the cells were incubated with rabbit anti-p-Parkin (Santa Cruz) and mouse anti-BCL-B (Abcam) antibodies at 4 °C overnight. The coverslips were incubated with secondary antibodies (FITC goat anti-rabbit IgG, 1:100, and CY3 goat anti-mouse IgG, 1:100, KPL) for 1 h at room temperature. After washing with PBS, the cells were stained with DAPI (1:2000, Sigma) for 5 min. The images were acquired under a laser scanning confocal microscope (Olympus, Japan).

### PicoGreen staining

The PicoGreen staining was performed with cells grown in six-well plates on glass cover slips. The mitochondrial DNA (mtDNA) staining in live cells was achieved by diluting the stock PicoGreen solution (Molecular Probes Inc., Oregon, USA) at 1 μl/ml directly into cell culture medium. The cells were incubated for 1 h. The cells were fixed in 4% paraformaldehyde and stained with DAPI (1:2000, Sigma). The images were acquired under a laser scanning confocal microscope (Olympus, Japan).

### Statistical analysis

All statistical tests were performed using SPSS 16.0 software. The differences between two groups were analyzed with Student’s t-tests. The differences among multiple groups were analyzed with one-way of variance. The data are presented as the mean ± SEM, and *P* < 0.05 was considered significant.

## Results

### Mitophagy is enhanced in parallel with increased apoptosis in primary hepatic stellate cells during the reversal of hepatic fibrosis

To evaluate apoptosis and mitophagy in HSCs during the process of the reversal of hepatic fibrosis, we established a model of hepatic fibrosis reversal. Hepatic fibrosis and HSC activation were assessed by a morphological analysis of H&E and Masson’s trichrome staining, immunohistochemistry and western blot assays of collagen I and α-smooth muscle actin (α-SMA). Hepatic fibrosis and HSC activation were clearly observed after 6 weeks of intraperitoneal injections of CCl_4_ and were reduced in a time-dependent manner after the withdrawal of CCl_4_ (Fig S1A–E). The hepatic damage was reduced over time after the withdrawal of CCl_4_ as assessed by the serum alanine aminotransferase (ALT) and aspartate aminotransferase (AST) levels (Fig S1F).

**Fig. 1:**
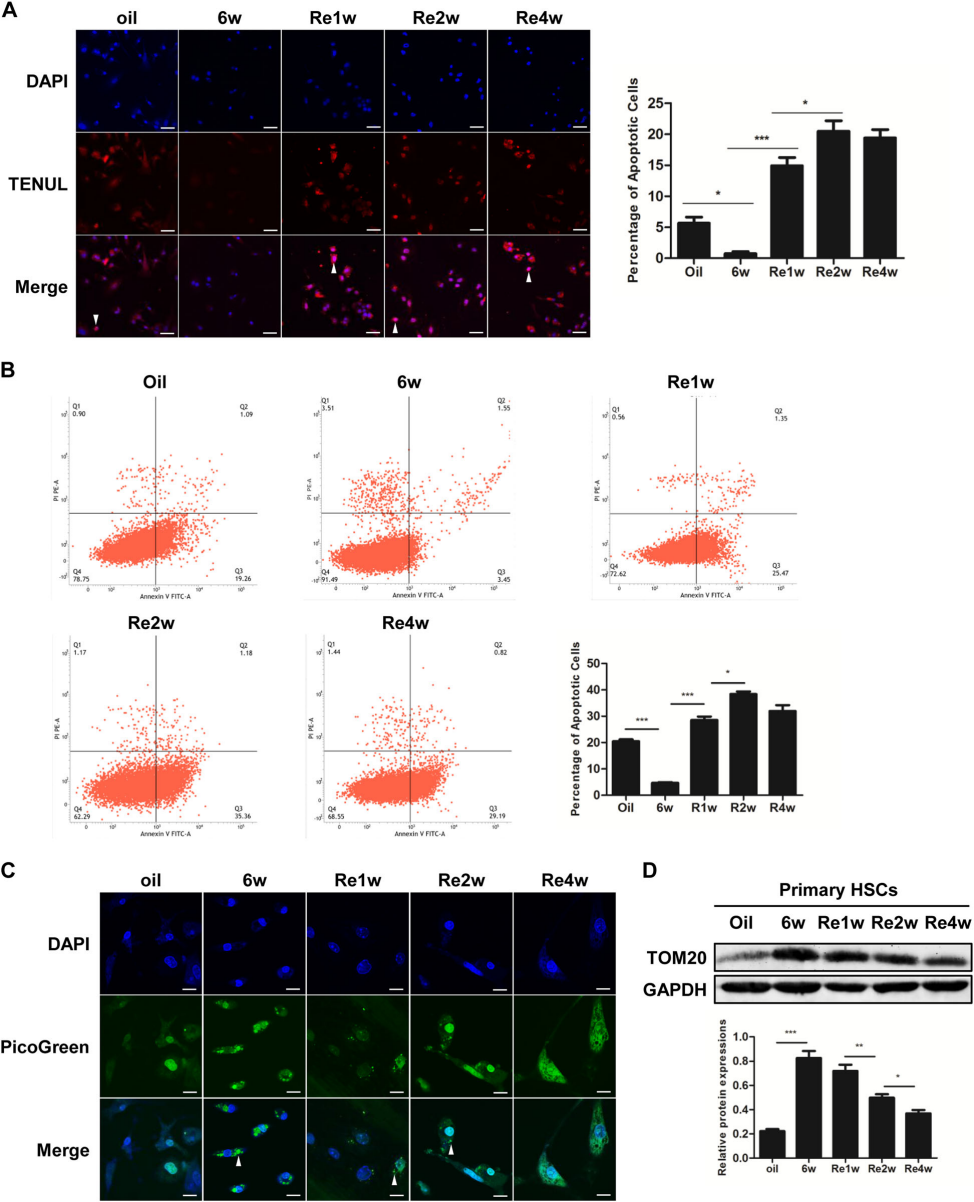
Mitophagy is enhanced in parallel with increased apoptosis in primary hepatic stellate cells during the reversal of hepatic fibrosis. **a** TUNEL staining of primary HSCs derived from the indicated mice; scale bar, 25 μm. Bar graph represents the mean ± SEM. *P < 0.05 and ***P < 0.001 vs. the indicated groups. **b** Annexin V-FITC/PI double-staining and flow cytometry analysis of primary HSCs; bar graph represents the mean ± SEM. *P < 0.05 and ***P < 0.001 vs. the indicated groups. **c** Mitochondrial DNA (mtDNA) measured by PicoGreen staining; scale bar, 10 μm. **d** Representative western blots of the mitochondrial membrane protein TOM20 with GAPDH serving as the internal reference

Apoptosis was analyzed by TUNEL staining and Annexin-V/propidium iodide double-staining assays of primary HSCs isolated from the mouse model. The apoptosis rate in the HSCs was decreased in the fibrotic mice after 6 weeks of CCl_4_ injections. The withdrawal of CCl_4_ resulted in an increase in HSC apoptosis, which peaked after 2 weeks of recovery (Figs. 1a, b). Mitophagy is the main method of the elimination of mitochondria^22^ and was evaluated by an analysis of the levels of mtDNA and proteins. PicoGreen can be used to specifically stain mtDNA^23^. The bright fluorescent spots observed in the cytoplasm were recognized as the typical staining pattern of mtDNA. The mtDNA and the expression of the mitochondrial protein TOM20 were increased by 6 weeks of the CCl_4_ treatment, indicating that mitophagy was reduced during hepatic fibrosis. The mtDNA and TOM20 levels decreased over the recovery time after the final injection of CCl_4,_ suggesting that mitophagic activity was enhanced during the regression of hepatic fibrosis. (Figs. 1c, d).

## Inhibition of mitophagy suppresses apoptosis in HSCs

**Fig. 2:**
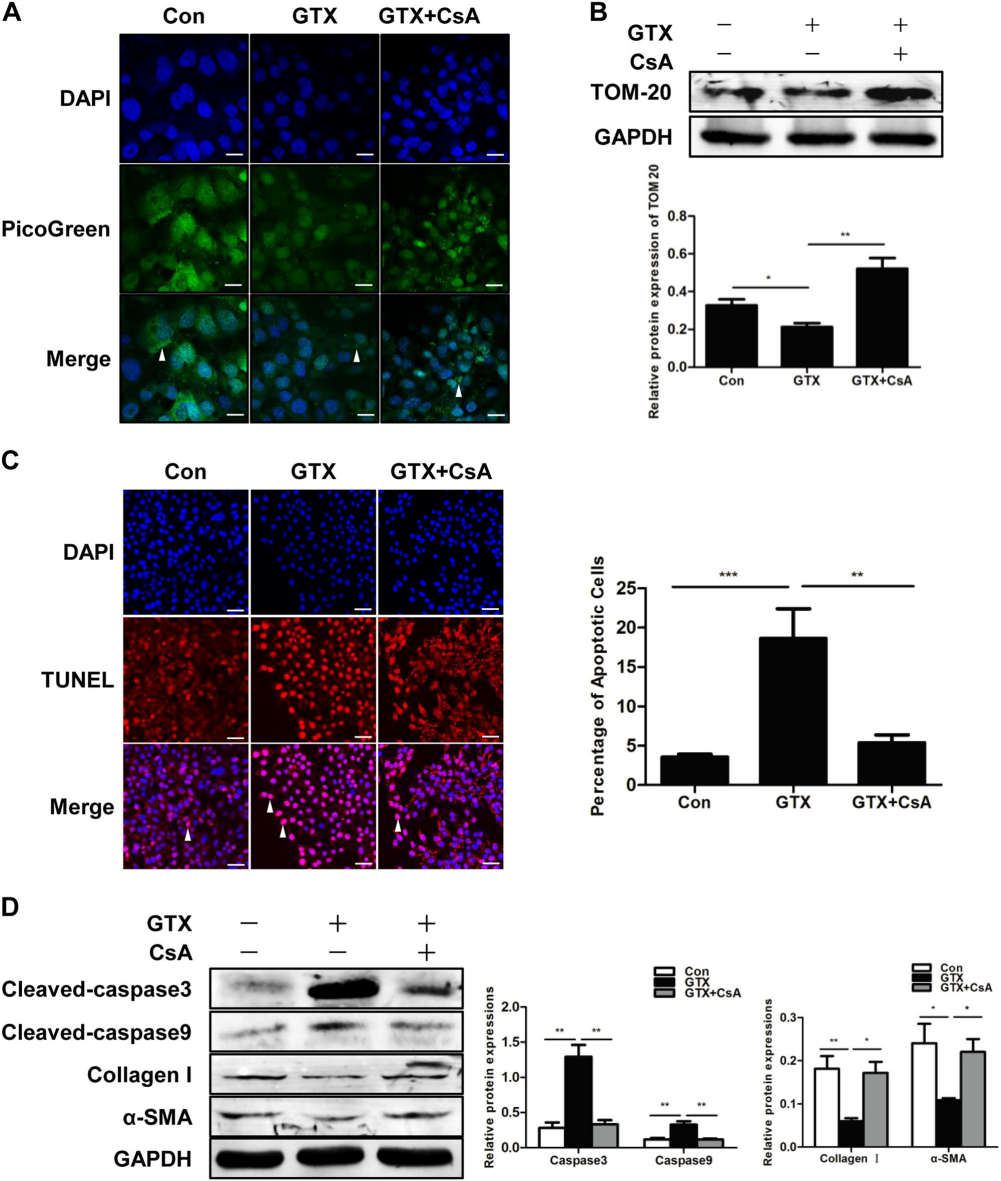
Inhibition of mitophagy suppresses apoptosis in HSCs. **a** Mitochondrial DNA (mtDNA) measured by PicoGreen staining in LX2 cells; Scale bar, 10 μm. **b** Representative western blots of TOM20 with GAPDH serving as the internal reference. Bar graph represents the mean ± SEM. ^*^P < 0.05 and **P < 0.01 vs. the indicated groups. **c** TUNEL staining of LX2 cells from the indicated groups; scale bar, 25 μm. Bar graph represents the mean ± SEM. **P < 0.01 and ***P < 0.001 vs. the indicated groups. **d** Representative western blots of cleaved caspase3, cleaved caspase9, collagen I and α-SMA. Bar graph represents the mean ± SEM of three different experiments. *P < 0.05 and **P < 0.01 vs. the indicated groups

To determine whether mitophagy plays a role in HSC apoptosis, we used cyclosporine A (CsA) to inhibit mitophagy. Then, we evaluated the effect on apoptosis induced by gliotoxin (GTX) in the human HSC cell line LX2. The treatment with CsA successfully inhibited mitophagy as the TOM20 and mtDNA levels were upregulated after the CsA treatment (Figs. 2a, b). The incubation with GTX caused an increase in the number of TUNEL-positive cells and higher levels of the active forms of caspase3 and caspase9, which were reduced by the inhibitor of mitophagy CsA (Figs. 2c, d). Furthermore, GTX inhibited LX2 activation, and the expression levels of collagen I and α-SMA were decreased in the GTX-treated cells. This phenotype was rescued by the inhibition of mitophagy (Fig. 2d).

## Activator of mitophagy induces apoptosis in HSCs

**Fig. 3:**
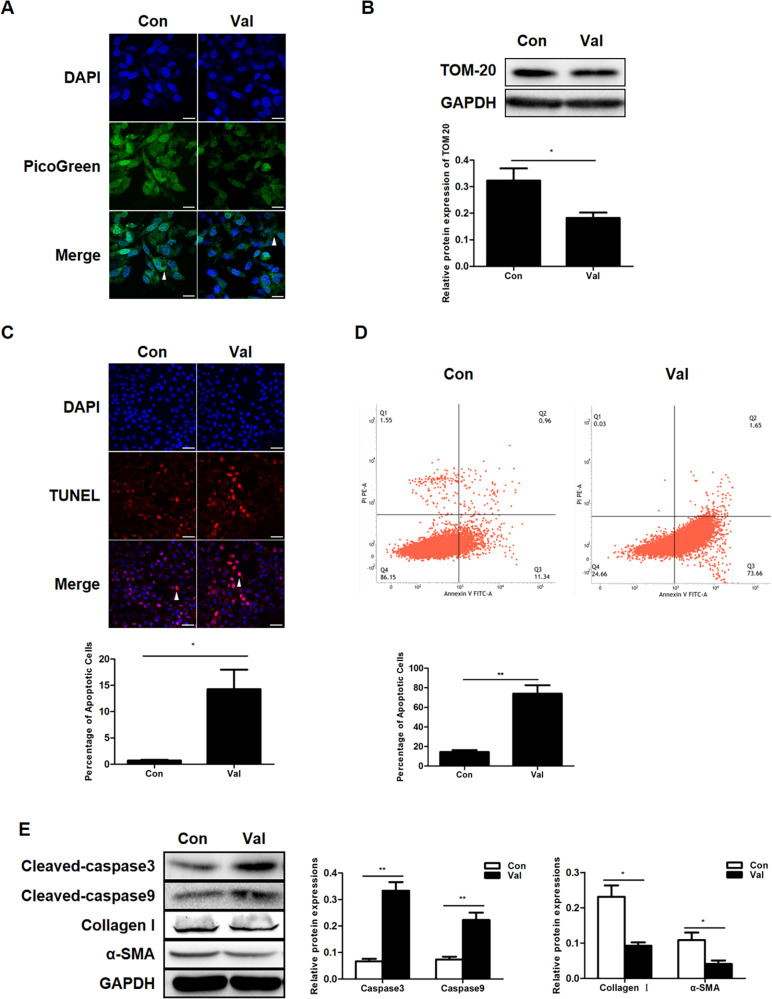
Activation of mitophagy induces apoptosis in HSCs. **a** Mitochondrial DNA (mtDNA) measured by PicoGreen staining in LX2 cells; scale bar, 10 μm. **b** Representative western blots of TOM20 with GAPDH serving as the internal reference. Bar graph represents the mean ± SEM. *P < 0.05 vs. the indicated groups. **c** TUNEL staining of LX2 cells from the indicated groups; scale bar, 25 μm. Bar graph represents the mean ± SEM. *P < 0.05 vs. the indicated groups. **d** Annexin V-FITC/PI double-staining and flow cytometry analysis of LX2 cells. Bar graph represents the mean ± SEM. **P < 0.01 vs. the indicated groups. **e** Representative western blots of cleaved caspase3, cleaved caspase9, collagen I and α-SMA. Bar graph represents the mean ± SEM of three different experiments. *P < 0.05 and **P < 0.01 vs. the indicated groups

The inducer of mitophagy valinomycin (Val) was used to further confirm the role of mitophagy in apoptosis. Val efficiently induced mitophagy as the content of the mtDNA and TOM20 was markedly reduced after the Val treatment (Figs. 3a, b). Val also caused an increase in the number of apoptotic cells as detected by the TUNEL and annexin V assays (Figs. 3c, d). Furthermore, the levels of activated caspase3 and caspase9 were upregulated (Fig. 3e). This induction of apoptosis was accompanied by a reduction in the expression of collagen I and α-SMA (Fig. 3e). These findings suggest that mitophagy plays a role in HSC apoptosis.

## Inhibition of mitophagy aggravates hepatic fibrosis in mice

**Fig. 4:**
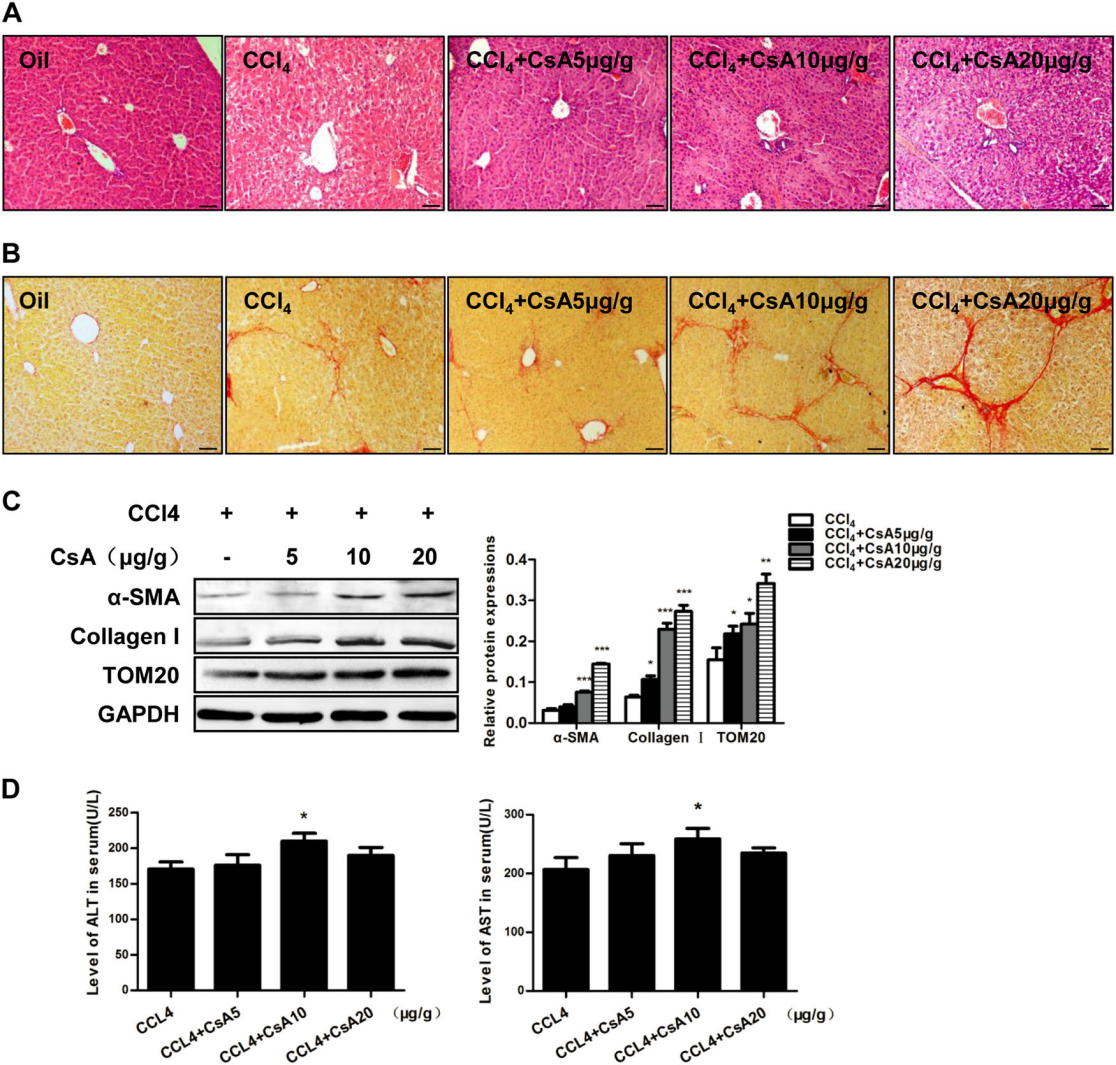
Inhibition of mitophagy aggravates hepatic fibrosis in mice. **a** H&E staining of liver sections from mice; *n* = 6 per group; scale bar, 50 μm. **b** Sirius red staining of collagen in mouse liver sections; *n* = 6 per group; scale bar, 50 μm. **c** Representative western blots of TOM20, collagen I and α-SMA. Bar graph represents the mean ± SEM of three different experiments. *P < 0.05; **P < 0.01; and ***P < 0.001 vs. the indicated groups. **d** Serum levels of ALT and AST in the indicated mice. Bar graph represents the mean ± SEM; *n* = 6 per group. *P < 0.05 vs. the indicated groups

To verify the anti-fibrotic effects of mitophagy in vivo, CsA (an inhibitor of mitophagy) was used in the mouse model of hepatic fibrosis at different concentrations (5, 10 and 20 μg/g). The CCl_4_-induced disorganization of hepatic lobules, collagen deposition and HSC activation were enhanced in a dose-dependent manner after the treatment with CsA (Figs. 4a–c). The serum levels of ALT and AST were increased after the injection of CsA at a dosage of 10 μg/g, suggesting that the liver injury was more severe in the mice treated with the mitophagy inhibitor (Fig. 4d).

## BCL-B inhibits mitophagy and apoptosis in HSCs

Our data demonstrate that mitophagy plays a role in HSC apoptosis. Therefore, the proteins involved in both mitophagy and apoptosis may be good candidates for mediating mitophagy-related apoptosis. The Bcl-2 family, which is a well-known superfamily of regulators of apoptosis, has also been reported to influence mitophagy^17^. We compared the mRNA levels of the members of the Bcl-2 family (including BCL-B, BCL-xL, Mcl-1, Bcl-W and A1) in LX2 cells after treatment with TGF-β and found that the TGF-β-induced expression was most obvious in BCL-2 (Fig S2A). The change in the expression of BCL-B was confirmed at the protein level. Both the withdrawal of CCl_4_ in the fibrotic mice and treatment with the mitophagy inducer Val in the LX2 cells resulted in a downregulation of BCL-B (Fig S2B, C). The treatment with CsA reversed the GTX-induced decrease in the BCL-B levels (Fig S2D). These findings suggest that BCL-B expression is inhibited during the process of mitophagy in HSCs and recovery of hepatic fibrosis.

**Fig. 5:**
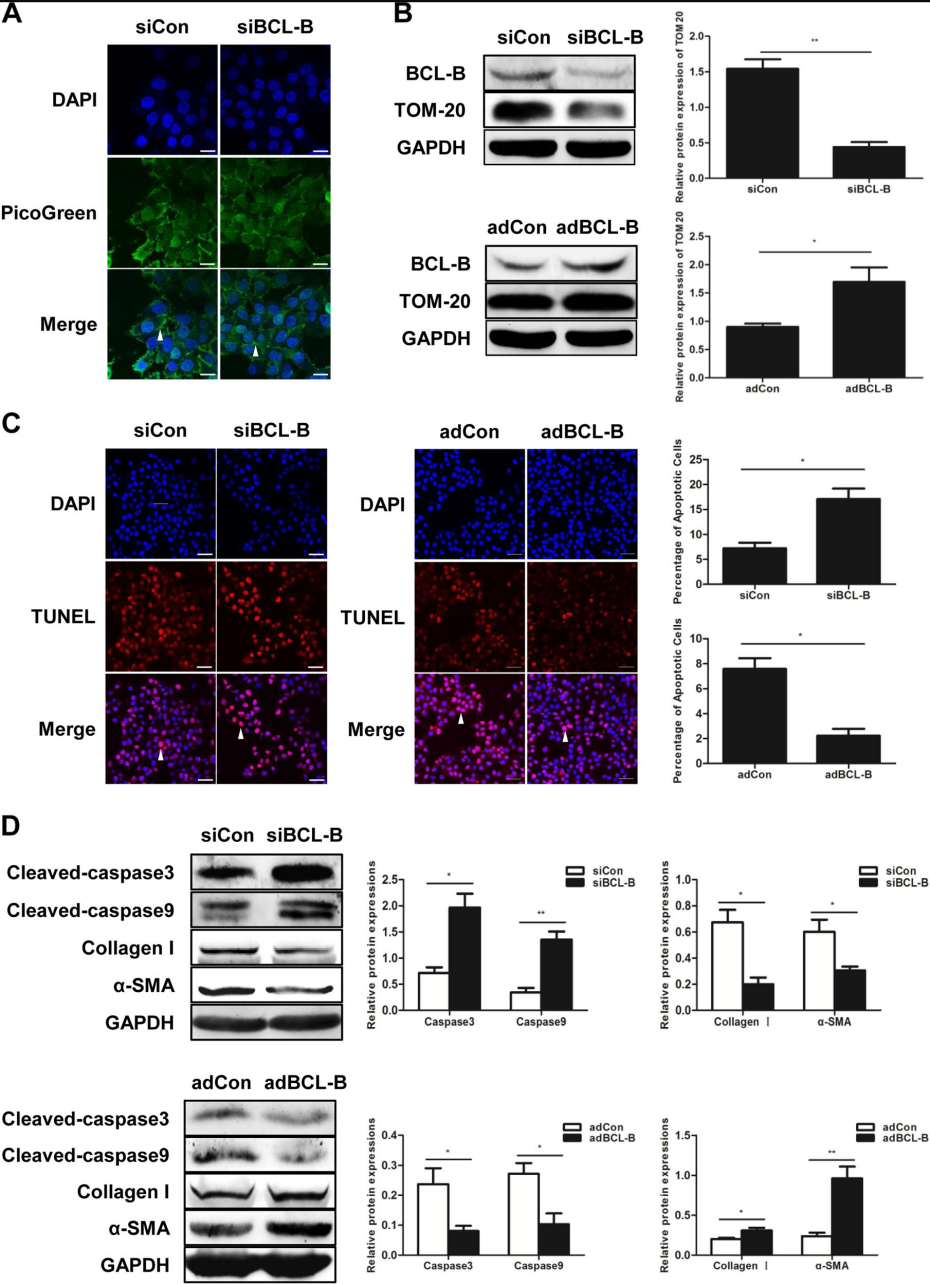
BCL-B inhibits mitophagy and apoptosis in HSCs. **a** Mitochondrial DNA (mtDNA) measured by PicoGreen staining in LX2 cells; scale bar, 10 μm. **b** Representative western blots of TOM20 and BCL-B. Bar graph represents the mean ± SEM of three different experiments. *P < 0.05 and **P < 0.01 vs. the indicated groups. **c** TUNEL staining of LX2 cells; scale bar, 25 μm. Bar graph represents the mean ± SEM. *P < 0.05 vs. the indicated groups. **d** Representative western blots of cleaved caspase3, cleaved caspase9, collagen I and α-SMA in LX2 cells. Bar graph represents the mean ± SEM of three different experiments. *P < 0.05 and **P < 0.01 vs. the indicated groups

To validate the effects of BCL-B on HSC apoptosis and mitophagy, BCL-B was either knocked down by small interfering RNA (siRNA) or overexpressed by infection with an adenoviral vector in LX2 cells. The knockdown of BCL-B led to a decrease in the mtDNA content and TOM20 expression, while the overexpression of BCL-B resulted in an increase in TOM20 expression (Figs. 5a, b), suggesting that BCL-B inhibited mitophagy in the LX2 cells. The number of TUNEL-positive cells and the expression levels of cleaved caspase3 and cleaved caspase9 were increased in the cells transfected with siRNA targeting BCL-B (siBCL-B) and decreased after the ectopic expression of BCL-B (Figs. 5c, d). Furthermore, the loss of BCL-B resulted in lower levels of collagen I and α-SMA, while the BCL-B overexpression upregulated these markers of HSC activation (Fig. 5d).

## BCL-B inhibits apoptosis by suppressing mitophagy

**Fig. 6:**
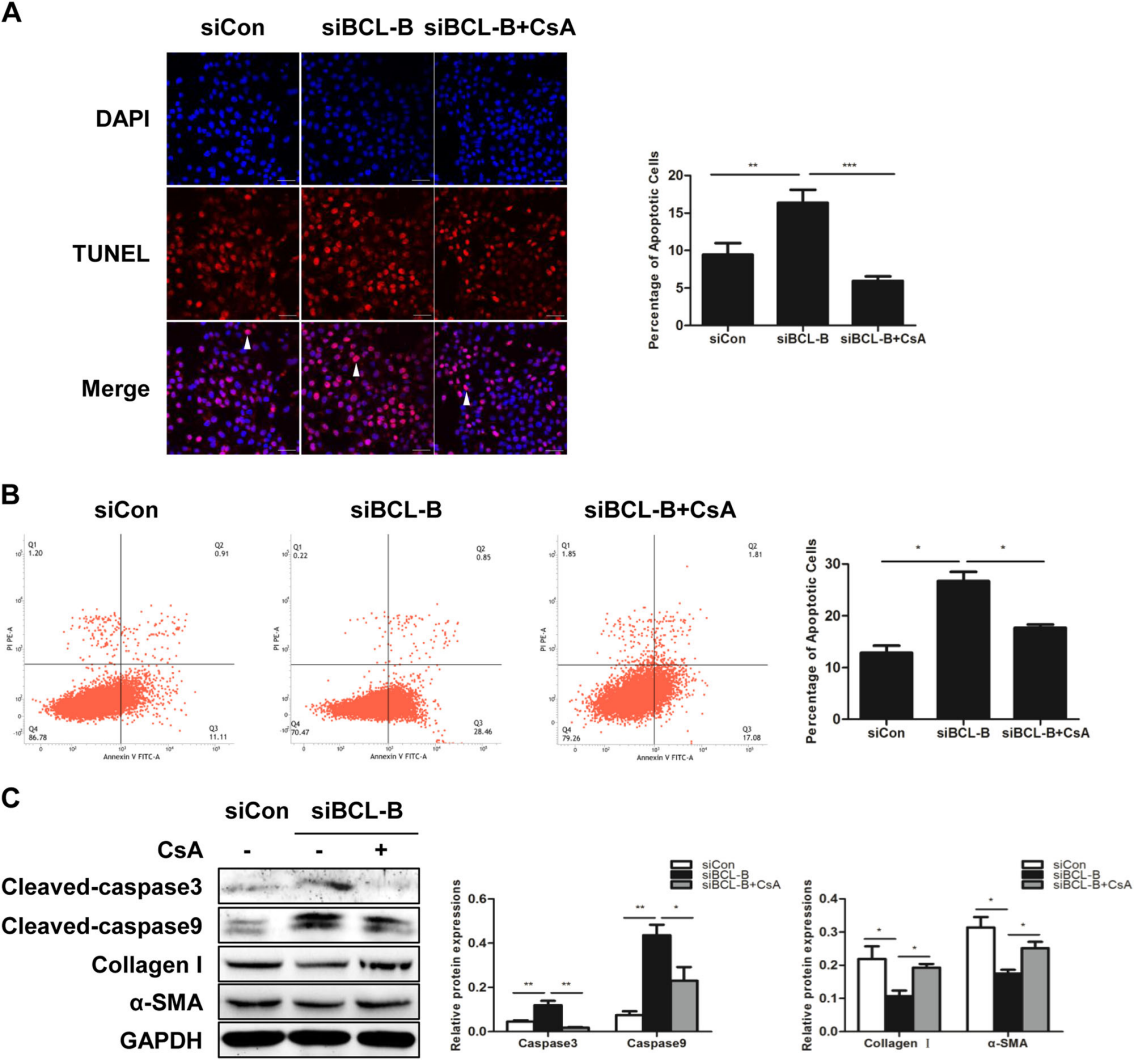
BCL-B inhibits apoptosis by suppressing mitophagy. **a** TUNEL staining of LX2 cells from the indicated groups; scale bar, 25 μm. Bar graph represents the mean ± SEM. **P < 0.01 and ***P < 0.001 vs. the indicated groups. **b** Annexin V-FITC/PI double-staining and flow cytometry analysis of LX2 cells. Bar graph represents the mean ± SEM. *P < 0.05 vs. the indicated groups. **c** Representative western blots of cleaved caspase3, cleaved caspase9, collagen I and α-SMA in LX2 cells. Bar graph represents the mean ± SEM of three different experiments. *P < 0.05 and **P < 0.01 vs. the indicated groups

We have shown that mitophagy plays a role in HSC apoptosis and that both mitophagy and apoptosis are inhibited by BCL-B. Subsequently, we speculated whether BCL-B regulates apoptosis through mitophagy. We used CsA to block mitophagy and investigated whether blocking mitophagy can reverse the apoptosis induced by the silencing of BCL-B. The apoptotic cell population identified by the TUNEL and Annexin V/PI double staining was significantly higher in the siBCL-B-transfected cells than that in the siCon-transfected cells. The increased apoptosis induced by the BCL-B knockdown was prevented by the CsA treatment (Figs. 6a, b). Consistently, the expression levels of cleaved caspase3 and cleaved caspase9 were elevated in response to the loss of BCL-B and reduced by CsA (Fig. 6c). Moreover, the impaired activation of HSCs that was attributed to the knockdown of BCL-B was restored by the CsA treatment (Fig. 6c).

## BCL-B inhibits mitophagy by binding phospho-Parkin

**Fig. 7:**
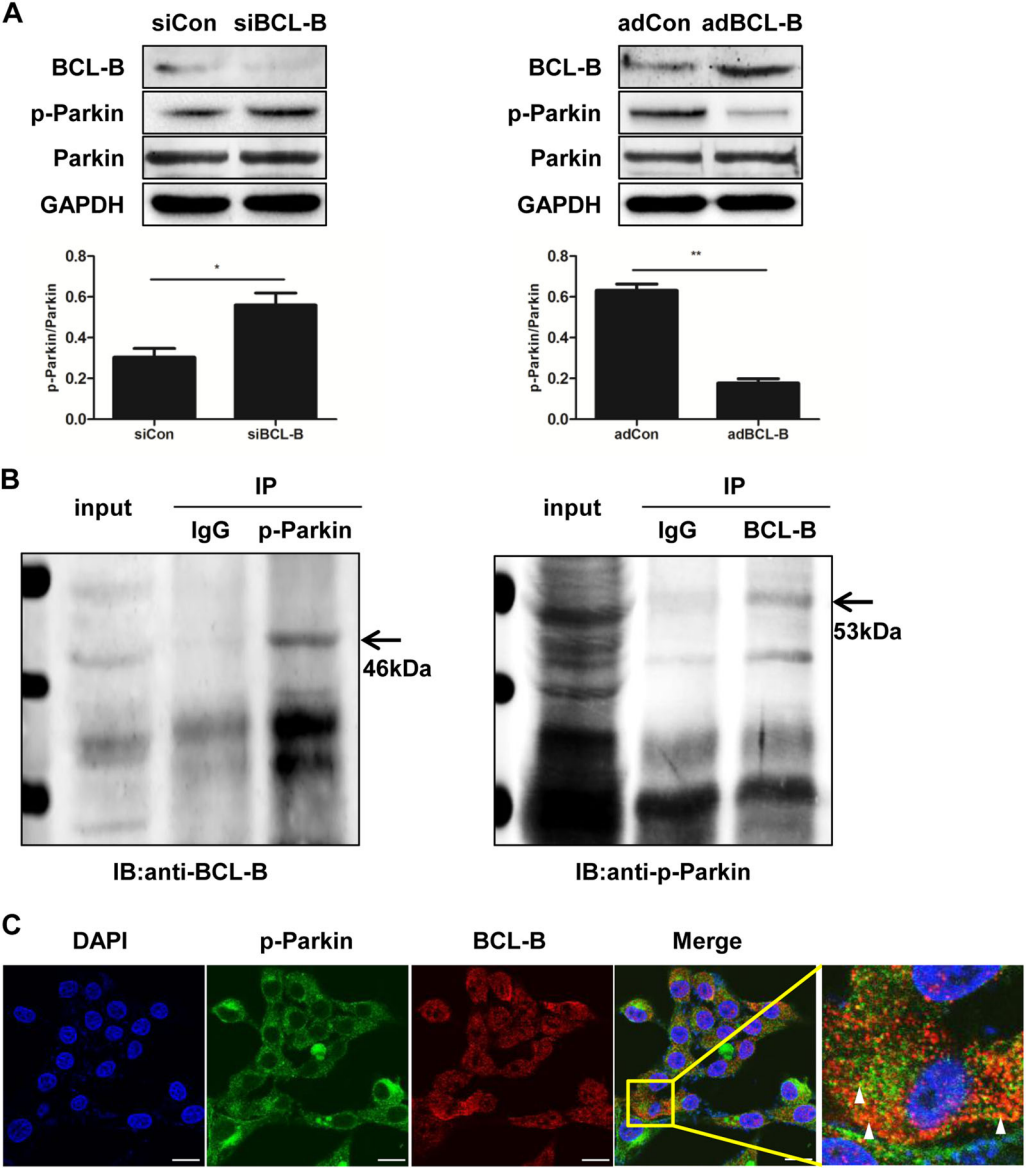
BCL-B inhibits mitophagy by binding phospho-Parkin. **a** Representative western blots of BCL-B, p-Parkin and Parkin in LX2 cells. Bar graph represents the mean ± SEM of three different experiments. *P < 0.05 and **P < 0.01 vs. the indicated groups. **b** Co-immunoprecipitation of the interaction between p-Parkin and BCL-B in LX2 cells. **c** Immunofluorescence staining of p-Parkin (green) and BCL-B (red) in LX2 cells. The nuclei were stained with DAPI (blue). Scale bar, 10 μm

The phosphorylation of Parkin by Pink1, which triggers its E3 ligase activity and the subsequent clearance of mitochondria, is a key event in mitophagy^24,25^. Therefore, we examined whether BCL-B influences the phosphorylation of Parkin. Neither the knockdown nor overexpression of BCL-B affected the expression of total Parkin. However, the loss of BCL-B led to an increase in the phosphorylation of Parkin, while the ectopic expression of BCL-B resulted in the opposite effect (Fig. 7a). As regulatory proteins usually directly interact with the target protein, we detected whether BCL-B can bind phospho-Parkin. The physical association between BCL-B and phospho-Parkin was confirmed by co-immunoprecipitation (Fig. 7b). The immunofluorescence double staining revealed a partial colocalization of BCL-B and phospho-Parkin in the LX2 cells (Fig. 7c).

## Discussion

Mitophagy is the main pathway by which injured mitochondria are cleared and plays a vital role in maintaining mitochondrial homeostasis, which is critical for apoptosis^7^. Additionally, the clearance of activated HSCs by apoptosis is critical for the reversibility of fibrosis. However, research investigating mitophagy in liver disease has mainly focused on hepatocytes, and research investigating mitophagy in HSCs and its effect on hepatic fibrosis is limited. In this study, we observed mitophagy in HSCs during the reversal of hepatic fibrosis for the first time. We found that mitophagy in HSCs was upregulated during the regression of hepatic fibrosis in parallel with increased apoptosis and promoted apoptosis in HSCs. Subsequently, we confirmed that BCL-B, which is a member of the BCL-2 family, is a regulator mediating mitophagy-related apoptosis. BCL-B inhibited mitophagy by binding and suppressing phospho-Parkin. Our findings identified a link between HSC mitophagy and hepatic fibrosis and provide a mechanism underlying the regulation of mitophagy-related apoptosis.

Previous studies investigating the effects of mitophagy on organ fibrosis have mainly focused on pulmonary fibrosis. PINK1-related mitophagy has been reported to inhibit fibrosis in the aging lung^4^. PINK-1 mediates PDGFR/PI3K/AKT activation, which contributes to the development of pulmonary fibrosis^26^. Furthermore, the age-related decline in mitophagy promotes pulmonary fibrosis^27^. These results indicate that mitophagy plays a protective role in pulmonary fibrosis. In liver fibrosis, melatonin-induced mitophagy displays an anti-fibrotic effect^28^. However, studies investigating the role of mitophagy in liver fibrosis have mainly focused on hepatocytes. Activated HSCs are the main source of collagen, which causes liver fibrosis, but few studies have investigated mitophagy in HSCs. We only found one study that mentioned that resveratrol induces mitophagy in HSCs^29^. However, the effect of mitophagy on HSC survival and liver fibrosis is still unknown. We found that mitophagy was enhanced during the reversal of hepatic fibrosis and that the inhibition of mitophagy aggregates hepatic fibrosis in mice. Our findings are consistent with previous studies suggesting that mitophagy protects against hepatic fibrosis.

Mitophagy is usually considered an antiapoptotic mechanism since it delays intrinsic apoptosis by limiting the release of pro-apoptotic factors from damaged mitochondria^30^. Mitophagy generally results in reduced apoptosis in various cell types^31–33^. However, under some circumstances, the selective targeting of mitochondria for autophagy can enhance apoptosis. Yee et al showed that the inhibition of mitophagy induced by PUMA, which is a p53-inducible BH3-only protein, dampened the apoptotic response^34^. Liu et al demonstrated that DRAM (damage-regulated autophagy modulator) translocated to mitochondria and induced mitophagy, leading to apoptosis in normal hepatocytes but not HCC cells^35^. In this study, we found that the decreased levels of mitochondrial DNAs and proteins due to enhanced mitophagy are consistent with low collagen synthesis in apoptotic HSCs and that mitophagy promotes apoptosis in HSCs during the reversal of hepatic fibrosis. It is possible that some factors regulating both mitophagy and apoptosis may be activated or inhibited during the regression of liver fibrosis.

Bcl-2 family proteins are well-known regulators of apoptosis that have been reported to suppress Parkin-related mitophagy^17^. We examined the TGF-β-induced mRNA expression levels of Bcl-2 family members with an emphasis on BCL-B, which is the most recently discovered member of the Bcl-2 family. BCL-B exerts either anti-apoptotic or pro-apoptotic activity depending on the cellular context^13^ and influences mitophagy in HeLa cells^17^. Thus, we speculated whether BCL-B regulates both HSC apoptosis and mitophagy during the regression of liver fibrosis.

We found that BCL-B inhibited both apoptosis and mitophagy in LX2 cells, suggesting that BCL-B might function as a regulator linking mitophagy and apoptosis during the regression of hepatic fibrosis. In conclusion, our data showed that mitophagy was enhanced during the reversal of hepatic fibrosis and promoted apoptosis in HSCs. BCL-B suppressed HSC apoptosis by inhibiting mitophagy by binding and suppressing phospho-Parkin. Here, for the first time, we provide evidence that BCL-B-related mitophagy is a new way of regulating HSC apoptosis during the regression of hepatic fibrosis.

## Supplementary information

Supplementary Materials
