# Author Correction: The role of the apoptosis-related protein BCL-B in the regulation of mitophagy in hepatic stellate cells during the regression of liver fibrosis

**DOI:** 10.1038/s12276-025-01515-z

**Published:** 2025-07-25

**Authors:** Qian Ding, Xiao-Li Xie, Miao-Miao Wang, Jie Yin, Jin-Mei Tian, Xiao-Yu Jiang, Di Zhang, Jing Han, Yun Bai, Zi-Jin Cui, Hui-Qing Jiang

**Affiliations:** Department of Gastroenterology, The Second Hospital of Hebei Medical University, Hebei Key Laboratory of Gastroenterology, Hebei Institute of Gastroenterology, Shijiazhuang, Hebei China

Correction to: *Experimental & Molecular Medicine* 10.1038/s12276-018-0199-6, published online 11 January 2019

After online publication of this article, the authors noticed an error in Fig. 3A, E. For Fig. 3A, the right panel (Val) needs to be corrected. As for Fig. 3E, the third panel (collagen I) requires revision. Other parts of this article remain unchanged.

The correct figure should have appeared as shown below.
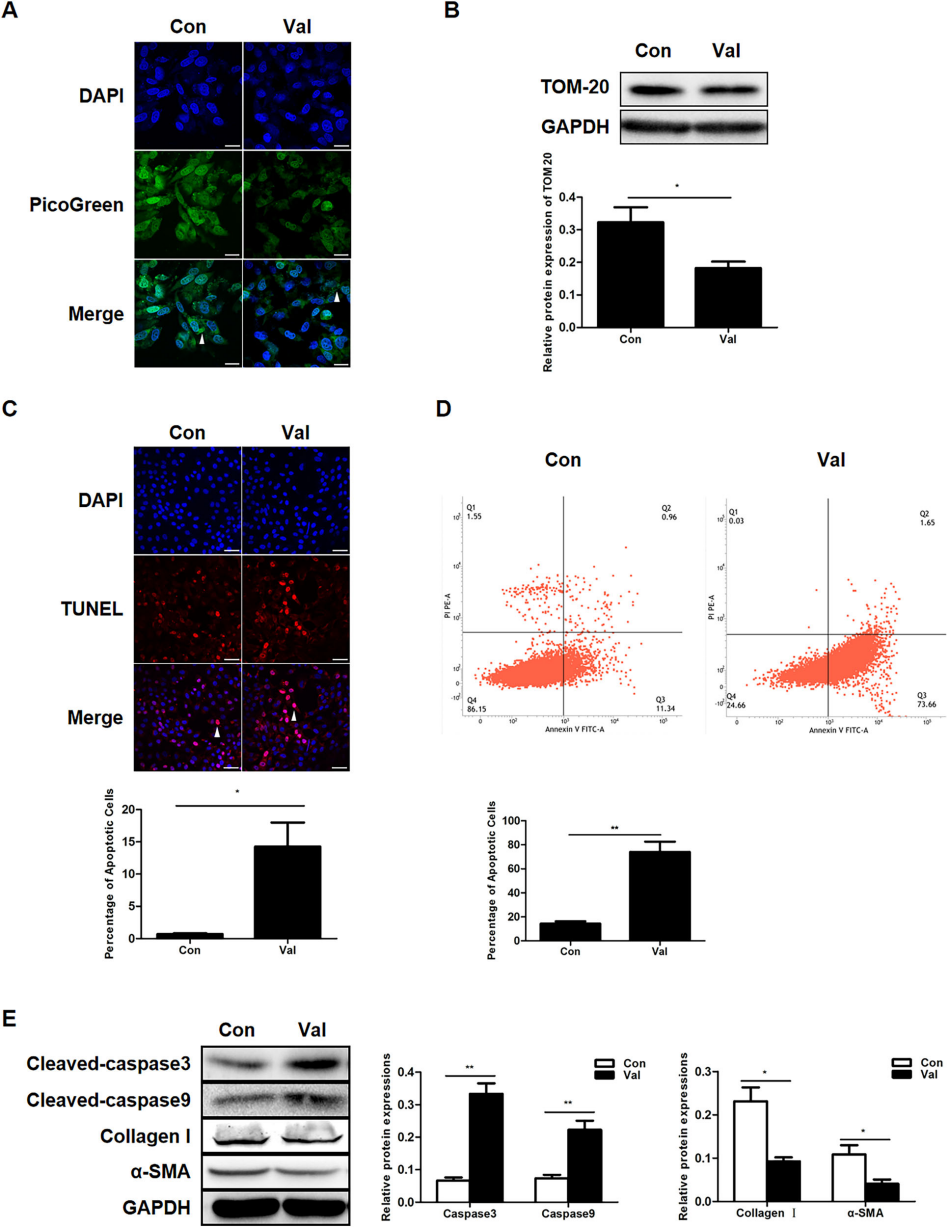


The authors apologize for any inconvenience caused.

The original article has been corrected.

