# Supplementary material for: The role of the apoptosis-related protein BCL-B in the regulation of mitophagy in hepatic stellate cells during the regression of liver fibrosis
Source: Exp Mol Med. 2019 Jan 11;51(1):1–13. doi: 10.1038/s12276-018-0199-6 (PMC6329697; doi:10.1038/s12276-018-0199-6)
Supplement: Supplementary file 1 — Supplementary Materials [file 12276_2018_199_MOESM1_ESM.docx]

**The Role of the Apoptosis-related Protein BCL-B in the Regulation of Mitophagy in Hepatic Stellate Cells** **during the Regression of Liver Fibrosis**

Qian Ding, PhD ^1^, Xiao-Li Xie, PhD ^1^, Miao-Miao Wang, PhD ^1^, Jie Yin, PhD^1^, Jin-Mei Tian, PhD ^1^, Xiao-Yu Jiang, PhD ^1^, Di Zhang, PhD ^1^, Jing Han, PhD ^1^, Yun Bai, PhD ^1^, Zi-Jin Cui, PhD ^1^, Hui-Qing Jiang, MD, PhD ^1^

**
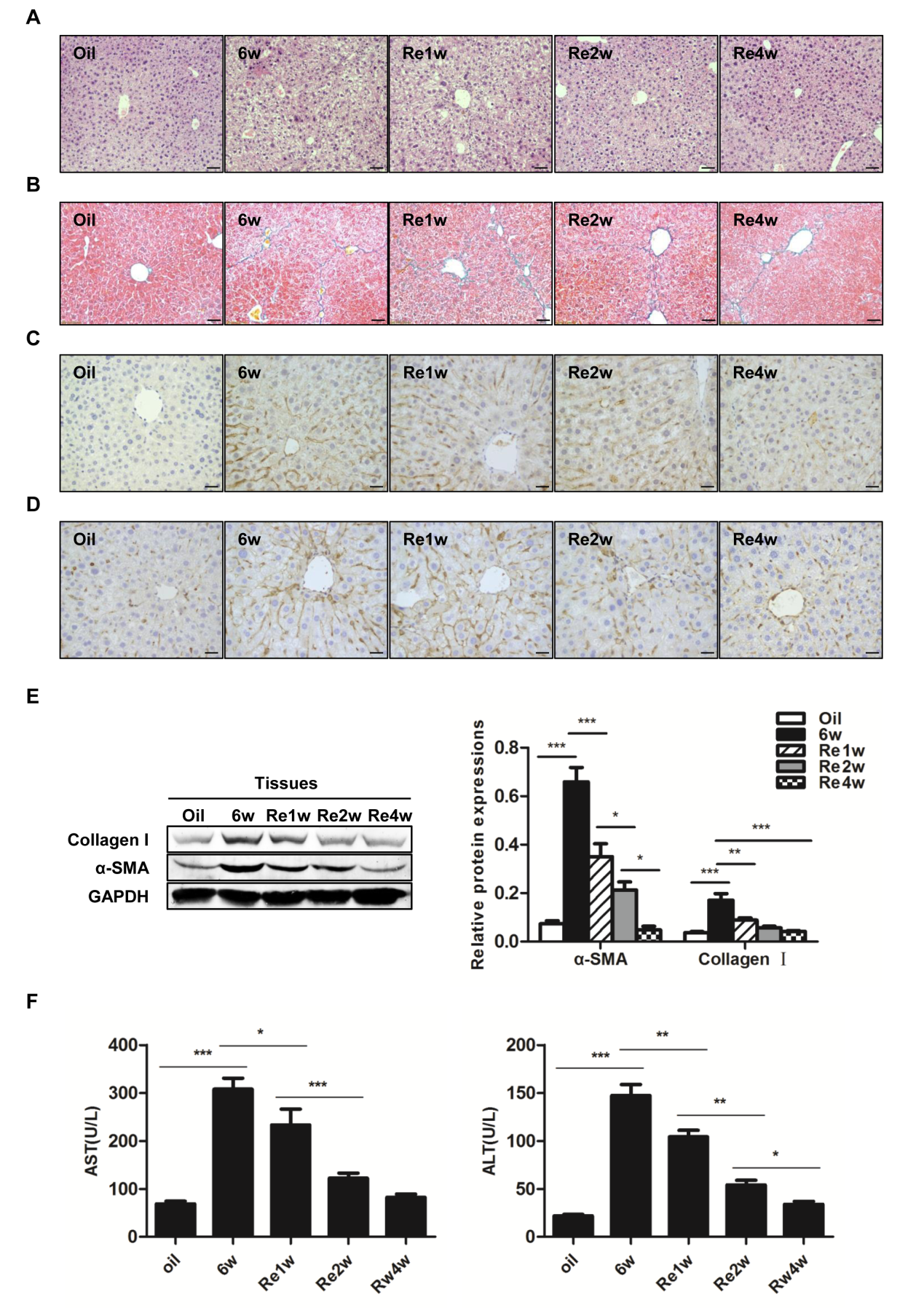
**

**Fig. S1.** Mouse model of hepatic fibrosis reversal.

(A) H&E staining of liver sections from mice, n=6 per group, scale bar, 50 μm. (B) Masson’s trichrome staining for collagen in the mouse liver, n=6 per group, scale bar, 50 μm. Immunohistochemistry staining for collagen I (C) and α-SMA (D) in mouse liver sections. n=6 per group, bar graph represents the mean ± SEM, scale bar, 25 μm. (E) Representative western blots of collagen I and α-SMA, bar graph represents the mean ± SEM; n = 6 per group, **P* <0.05, ***P* <0.01, ****P* <0.001 vs. the indicated groups. (F) Serum levels of ALT and AST in the mice. Bar graph represents the mean ± SEM; n = 6 per group, **P* <0.05, ***P* <0.01, ****P* <0.001 vs. the indicated groups.


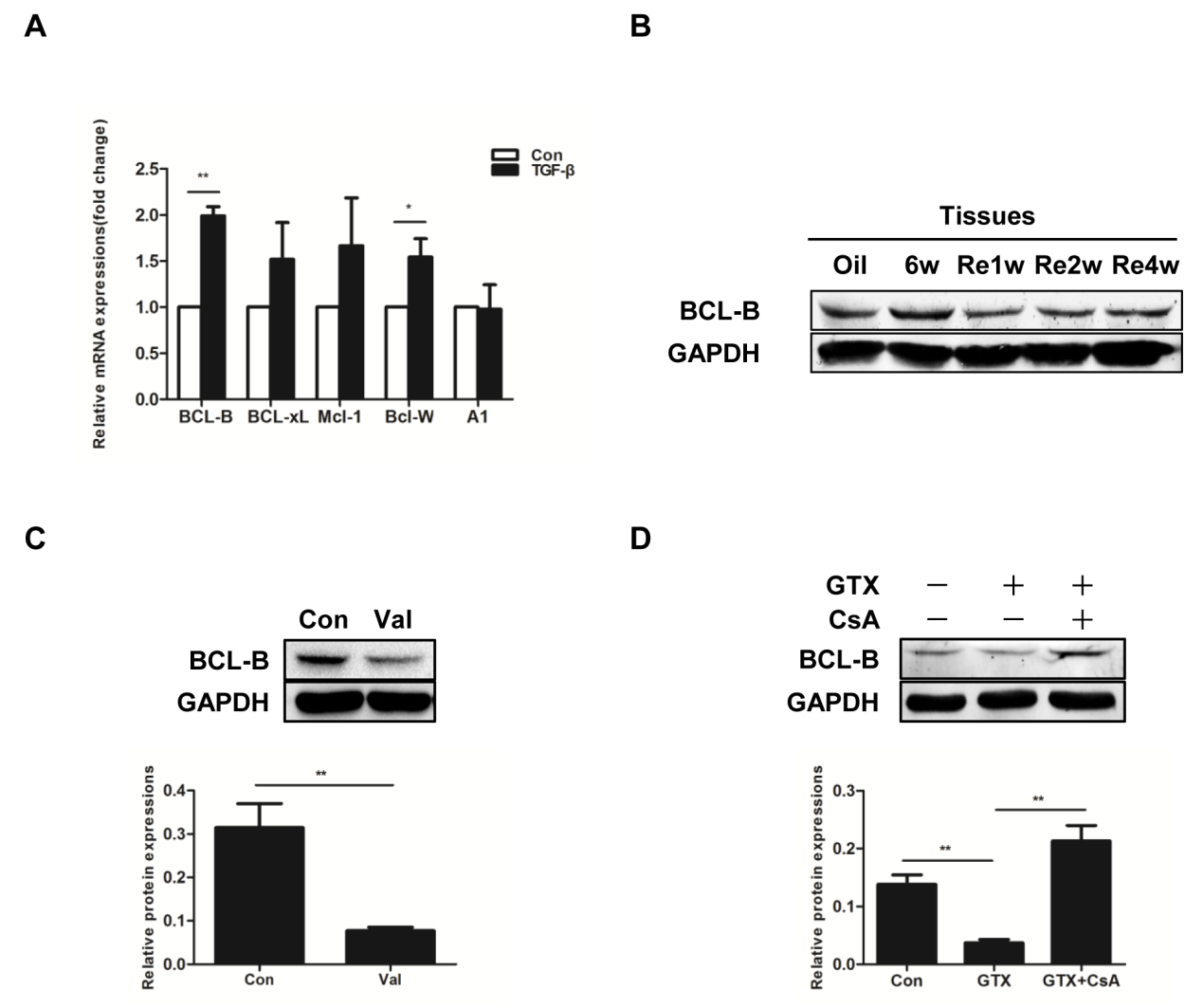


**Fig. S2**. Expression levels of BCL-B. (A) mRNA levels of the members of the BCL-2 family in LX2. Bar graph represents the mean ± SEM from three independent experiments, **P*<0.05, ***P*<0.01 vs. indicated groups. (B) Representative western blots of BCL-B from lysate isolated from mouse liver tissues. (C) and (D) Representative western blots of BCL-B in LX2. Bar graph represents the mean ± SEM from at least 3 independent experiments. ***P* <0.01 vs. the indicated groups.
